# Supplementary material for: Configurations of Adult Attachment, Indicators of Mental Health and Adverse Childhood Experiences in Women: A Cross-Sectional Study
Source: Int J Environ Res Public Health. 2021 Dec 19;18(24):13385. doi: 10.3390/ijerph182413385 (PMC8707459; doi:10.3390/ijerph182413385)
Supplement: Supplementary file 1 [file ijerph-18-13385-s001.zip › ijerph-1486449-supplementary.pdf]

International Journal of
Environmental Research and Public Health

Supplementary Materials: Table S1: Acronyms List

Table S1. Acronyms List.

	Acronym	Meaning
	ACE	Adverse Childhood Experience
Attachment Variables	FRA	Fear of Rejection or Abandonment
	DC	Desire for Closeness
	PI	Preference for Independence
Mental Health Indicators	SWL	Satisfaction With Life
	PA	Positive Affect
	NA	Negative Affect
	GSI	Global Severity Index of psychopathology
Subject Groups	1-BBB	Below the median in FRA, Below the median in DC, Below the median in PI
	2-BBA	Below the median in FRA, Below the median in DC, Above the median in PI
	3-BAB	Below the median in FRA, Above the median in DC, Below the median in PI
	4-BAA	Below the median in FRA, Above the median in DC, Above the median in PI
	5-ABB	Above the median in FRA, Below the median in DC, Below the median in PI
	6-ABA	Above the median in FRA, Below the median in DC, Above the median in PI
	7-AAB	Above the median in FRA, Above the median in DC, Below the median in PI
	8-AAA	Above the median in FRA, Above the median in DC, Above the median in PI
